# Neural Correlates of Facial Emotion Recognition in Non-help-seeking University Students With Ultra-High Risk for Psychosis

**DOI:** 10.3389/fpsyg.2022.812208

**Published:** 2022-06-09

**Authors:** Jiaojiao Hou, Simon Schmitt, Xudong Zhao, Jiayi Wang, Jianxing Chen, Ziyu Mao, Ansi Qi, Zheng Lu, Tilo Kircher, Yunbo Yang, Jingyu Shi

**Affiliations:** ^1^Department of Psychosomatic Medicine, Tongji University School of Medicine, Shanghai East Hospital, Shanghai, China; ^2^Department of Psychiatry, University of Marburg, Marburg, Germany; ^3^Center for Mind, Brain and Behavior, University of Marburg, Marburg, Germany; ^4^Hannover Medical School, Clinics for Psychiatry, Social Psychiatry and Psychotherapy, Hannover, Germany; ^5^Shanghai Pudong New Area Mental Health Center, Tongji University School of Medicine, Shanghai, China; ^6^Tongji University School of Medicine, Shanghai, China; ^7^Ruijin Hospital Luwan Branch, Shanghai Jiao Tong University, School of Medicine, Shanghai, China; ^8^Department of Medical Psychology, Shanghai General Hospital, Shanghai Jiao Tong University, Shanghai, China; ^9^Department of Psychiatry, Shanghai Tongji Hospital, Tongji University School of Medicine, Shanghai, China; ^10^Department of Psychiatry, Shanghai Mental Health Center, Shanghai Jiao Tong University School of Medicine, Shanghai, China; ^11^Division of Medical Humanities and Behavioral Sciences, Tongji University School of Medicine, Shanghai, China

**Keywords:** clinical high risk for psychosis, facial emotion recognition, superior temporal gyrus, functional magnetic resonance imaging (fMRI), social cognition, prodromal psychosis

## Abstract

**Background:**

Since the introduction of the neurodevelopmental perspective of schizophrenia research on individuals at ultra-high risk for psychosis (UHR) has gained increasing interest, aiming at early detection and intervention. Results from fMRI studies investigating behavioral and brain functional changes in UHR during facial emotion recognition, an essential component of social cognition, showed heterogenous results, probably due clinical diversity across these investigations. This fMRI study investigated emotion recognition in a sub-group of the UHR spectrum, namely non-help-seeking, drug-naïve UHR with high cognitive functioning to reveal the neurofunctional underpinnings of their social functioning in comparison to healthy controls.

**Methods:**

Two large cohorts of students from an elite University (*n*_1_ = 4,040, *n*_2_ = 4,364) were screened firstly with the Prodromal Questionnaires and by surpassing predefined cut-offs then interviewed with the semi-structured Interview for Psychosis-Risk Syndromes to verify their UHR status. Twenty-one identified non-help-seeking UHR and 23 non-UHR control subjects were scanned with functional magnetic resonance imaging while classifying emotions (i.e., neutral, happy, disgust and fear) in a facial emotion recognition task.

**Results:**

Behaviorally, no group differences were found concerning accuracy, reaction times, sensitivity or specificity, except that non-help-seeking UHR showed higher specificity when recognizing neutral facial expressions. In comparison to healthy non-UHR controls, non-help-seeking UHR showed generally higher activation in the superior temporal and left Heschl's gyrus as well as in the somatosensory, insular and midcingulate cortex than the control subjects during the entire recognition task regardless of the emotion categories. In an exploratory analysis, in the non-help-seeking UHR group, functional activity in the left superior temporal gyrus was significantly correlated with deficits in the ability to experience emotions at uncorrected statistical thresholds.

**Conclusions:**

Compared to healthy controls, non-help-seeking UHR show no behavioral deficits during facial emotion recognition, but functional hyperactivities in brain regions associated with this cognitive process. Our study may inspire future early intervention and provide loci for treatment using neural stimulation.

## Introduction

In the past three decades, the concept of potentially prodromal prepsychotic symptoms has been developed to prospectively identify people who are at high risk for psychosis (Fusar-Poli et al., 2013). According to the well-established definition of the ultra-high risk concept (Yung et al., 2004; Fusar-Poli et al., 2013; Yung and Nelson, 2013), UHR (individuals at ultra-high risk for psychosis) are fulfilling at least one of the following criteria: attenuated psychotic symptoms (APS), brief limited intermittent psychotic symptoms (BLIPS) and genetic risk and deterioration syndrome (GRS; Yung et al., 2008; Fusar-Poli et al., 2015a). UHR status is a strong predictor of the development of psychotic disorders, a meta-analysis estimates the transition risk of 18% after 6 months of follow-up and of 36% after 3 years of follow-up (Fusar-Poli et al., 2012a). The range of functional and clinical characteristics of UHR include, among others, disorganized speech, unusual thought content, perceptual abnormalities, paranoid ideation and deficits in social cognition (Fusar-Poli et al., 2013).

A key component in social cognition is facial emotional recognition, which is the ability to accurately identify universal signals of emotional disposition in facial expressions. This helps us to emotionally connect with others and effectively communicate with them (Ekman, 1993; Fusar-Poli et al., 2009). Deficits in facial emotion recognition have been repeatedly demonstrated in patients with schizophrenia (Sachs et al., 2004; Martin et al., 2005; Barkl et al., 2014; Gabay et al., 2015). Two meta-analyses investigated facial emotion recognition in UHR on a behavioral level (Lee et al., 2015; van Donkersgoed et al., 2015). Both found that UHR perform moderately poorer than controls. However, the authors (van Donkersgoed et al., 2015) noted substantial heterogeneity in the outcomes of the selected studies probably due to the clinical and methodological diversity across the selected studies. Therefore, these results must be interpreted with caution.

On the neurofunctional level, facial expression recognition is correlated with brain activation in the fronto-temporo-occipital network and limbic structures including the amygdala, especially in the bilateral superior temporal gyrus (STG) in healthy population (Johnston et al., 2005; Sliwinska and Pitcher, 2018). Parallel to their impaired behavioral performance when processing facial emotions, patients with schizophrenia showed attenuated neural activity in the fronto-temporo-occipital network and limbic structures when recognizing different facial emotions irrespective of emotion types versus control condition contrast (Taylor et al., 2012; Jáni and Kašpárek, 2018). Systematic reviews on emotional processing in people at clinical high-risk for psychosis (Kozhuharova et al., 2020; Lukow et al., 2021) found convergence across studies that reported increased activation in the cingulate and frontal cortices. However, the overall results of all studies were again inconclusive probably due to their heterogeneity in both imaging paradigms as well as samples.

Remarkably, some studies reported increased functional activity to neutral conditions in the inferior frontal gyrus (Seiferth et al., 2008; Modinos et al., 2015), the left temporal pole and bilateral posterior cingulate cortex (van der Velde et al., 2015), the thalamus (Seiferth et al., 2008) and in the insula (Modinos et al., 2015). Due the thalamus is implicated in controlling emotional attention (Phillips et al., 2003) and the insula plays a crucial role in the evaluative, experiential and expressive processing of internally generated emotions (Craig, 2009), their increased responses to neutral conditions were interpreted as a potential neural hypersensitivity to affectively irrelevant stimuli that may be related to aberrant salience processing (Kozhuharova et al., 2020).

There are many factors that could potentially explain the aforementioned heterogeneity of study results: 1. UHR are more likely to show DSM Axis I comorbid disorders like major depressive disorder (Fusar-Poli et al., 2012b, 2017) which is associated with impairments in emotion recognition (Dalili et al., 2015). 2. Similarly, some UHR with comorbid affective and/or anxiety disorders could use antidepressant pharmacotherapy that also might affect emotion recognition (Harmer et al., 2011, 2013), potentially via modulations of amygdala responses to emotional facial expressions (Murphy et al., 2009). 3. In schizophrenia patients, it has been demonstrated that facial affect perception accuracy did not significantly differ between high-functioning patients and non-psychiatric healthy controls but was significantly lower in low-functioning schizophrenia patients compared to the two other aforementioned groups (Karpouzian et al., 2017). A similar effect might be found in UHR which could be another factor causing heterogenous results in previous studies. 4. There is strong evidence that genetic risk for schizophrenia which is elevated in UHR (see GRS criterion) is associated with facial emotion recognition deficits (Martin et al., 2020). 5. Another factor causing clinical heterogeneity within UHR samples is that UHR need to fulfill only one from three potentially concurrent criteria that comprise a wide symptomatic range. 6. Last, within the spectrum of UHR, a subgroup has been identified that consists of non-help seeking UHR (NHS-UHR) who differ from help-seeking UHR with respect to sociodemographic and clinical factors (Falkenberg et al., 2015; Fusar-Poli et al., 2015b). While the overall level of functioning of NHS-UHR is higher than that of clinical UHR, psychosis-risk symptoms as well as psychosis-risk criterions each significantly predicted functional deficits in NHS-UHR (Schultze-Lutter et al., 2018). However, the majority of research have only investigated help-seeking UHR (Amminger et al., 2012; Rietdijk et al., 2013; Thompson et al., 2015).

In order to disentangle these potentially confounding effects, in this study, we investigated NHS-UHR with no history of psychiatric comorbidities or psychopharmacological therapy and no parents with history of psychotic disorders. That there has not been lots of research on NHS-UHR has certainly many causes, e.g., they are more difficult to recruit because their prevalence is relatively smaller, because of their better functioning it is less likely that they will contact clinicians that often help with the recruitment and, importantly, the practical utility of ultra-high risk criteria for psychosis prediction is questioned by some researchers when applied outside clinical samples (Fusar-Poli, 2017). Remarkably, some widespread tools that assess clinical high-risk for psychosis, e.g., the CAARMS (Comprehensive Assessment of the At-Risk Mental State; Yung et al., 2005), require a drop in functioning regardless of which of the three UHR criteria (APS, BLIPS, GRS) are fulfilled, making it impossible to use for the screening of high-functioning UHR. The prevalence of NHS-UHR is estimated to range between 1.3% in the general population (Schimmelmann et al., 2015) to 2.4% in young adults (Schultze-Lutter et al., 2018) when the diagnostic criteria from the SIPS [“Structured Interview for Psychosis-Risk Syndromes” (McGlashan et al., 2001)] are applied and only 1.03% when utilizing the criteria recommended in the Guidance project of the “European Psychiatric Association” [Schultze-Lutter et al., 2015 i.e., APS syndrome, BIPS syndrome, Cognitive Disturbances (COGDIS)]. Their general functioning shows the highest negative correlations with the symptoms avolition and unusual thought content among various psychopathological symptoms (Ayoub et al., 2020).

In line with findings from Karpouzian et al. (2017) on high-functioning schizophrenia patients, we hypothesize that, on a behavioral level, NHS-UHR and a healthy control group (CG) do not differ significantly from each other. Second, we expect increased brain functional responses during facial emotion recognition in NHS-UHR relative to a CG. We expect to find these hyperactivations in the cingulate and fronto-temporal cortices, especially in the precentral cortex and the STG that have shown hyperactivations during this task in high-functioning schizophrenia patients as well as in UHR (Kozhuharova et al., 2020; Lukow et al., 2021). Next, we expected increased responses in the insula, the thalamus, temporal poles, cingulate cortex and inferior frontal gyrus in NHS-UHR compared to the CG during the processing of neutral facial emotional stimuli. In our previous brain morphological study (Hou et al., 2020), we investigated changes in cortical complexity, a biomarker measuring cortical folding (Di Ieva et al., 2013), in NHS-UHR and found cortical complexity in NHS-UHR in the STG to be reduced compared to a healthy CG. We therefore investigated in an exploratory analysis whether potential changed functional activity in the STG during facial emotion recognition in UHR is correlated with positive or negative prodromal symptoms.

## Methods

### Recruitment Procedure

Undergraduate students from two consecutive school years from Tongji University, Shanghai, an elite University with an acceptance rate of < 0.2%, were screened using a two-staged protocol. In the first step, the Prodromal Questionnaire (PQ-16; Ising et al., 2012) was used for the first school year and the Prodromal Questionnaire-brief version (PQ-B; Loewy et al., 2011) for the second school year in order to preselect participants with prodromal symptoms. A systematic review comparing different versions of the PQ concluded that both the PQ-B as well as the PQ-16 are both able to accurately identify UHR (Savill et al., 2018).

The 16-item version of the Prodromal Questionnaire (PQ-16) is a self-report questionnaire used to screen individuals with a high risk of psychosis by measuring e.g., perceptual abnormalities/hallucinations, unusual thought content/delusional ideas and negative symptoms (Ising et al., 2012). According to the recommendations from a previous study that also investigated Chinese NHS-UHR who were college students (Su et al., 2015), we set the cut-off value for the identification of individuals that should be included in the second stage of the screening protocol to 9. With this cut-off value, the Chinese version of the PQ-16 was able to detect psychosis risk with a sensitivity of 68% and a specificity of 73% (Su et al., 2015).

The PQ-B (Loewy et al., 2011) consists of 21-items to screen ultra-high risk psychosis. If the scores of individuals exceeded the cut-off score 24, participants were included in the second stage of the screening protocol. The Chinese translation of the PQ-B shows both high sensitivity (82%) and specificity (46.8%) in the Chinese population (Xu et al., 2016).

The second stage of the screening consisted of the SIPS interview (Miller et al., 2003) and the GAF (Global Assessment of Functioning; Endicott et al., 1976) which were both assessed by trained psychiatrists. The semi-structured SIPS interview is a validated diagnostic tool for the identification of an ultra-high-risk for psychosis (Fusar-Poli et al., 2016). Its Chinese version has evidenced good reliability and validity (Zheng et al., 2012). The SIPS aims to assess the severity of positive (e.g., grandiose ideas, perceptual abnormalities/hallucinations and disorganized communication), negative (e.g., social anhedonia, avolition, expression of emotion, experience of emotions and self), disorganized and general symptoms (Chen et al., 2014).

Additionally, the Global Assessment of Functioning (GAF; Endicott et al., 1976) was applied by trained psychiatrists during the interview which is a rating scale that describes the participants' social, psychological and occupational functioning. Its values range from 0 to 100 and higher scores on the GAF indicate higher global functioning. A meta-analysis on functioning levels of people at high risk on psychosis found a mean GAF score of 50 for UHR, 79 for healthy controls and 45 for psychosis patients (Fusar-Poli et al., 2015b). Participants' GAF-score had to be above 60, otherwise they dropped out of the study.

If participants fulfilled the SIPS' criteria for being in a prodromal phase of psychosis, they were invited to undergo an MRI scan during the following 2 months. Participants who fulfilled one of the following criteria were excluded from further screening: GAF < 61, current or previous psychiatric disease, parents with current or previous psychotic disorder, i.e., schizophrenia, schizoaffective disorder or bipolar disorder, history of a disease or operation that could impair brain function or structure (e.g., severe CNS trauma, meningitis, cancer, several autoimmune diseases like lupus), drug abuse, receiving any neuropsychiatric treatment and self-reported inability to undergo an MRI-examination (e.g., claustrophobia, inability to lie still, epilepsy, pregnancy, MRI-incompatible metal implants).The CG were randomly selected from the remaining participants that did not exceed cut-off values from either PQ-16 or PQ-B in the first stage of the screening.

All participants provided informed consent before the experiment and received monetary reward after the completion of the study. The study protocol was approved by the ethics committee of the Institutional Review Board of Tongji University (No: 2019tjdx264).

### MRI Data Acquisition and MRI Data Preprocessing

MRI data were acquired using a 3.0 Tesla MRI scanner (GE MR750). Functional data were collected using an EPI sequence (flip angle = 90°, TR = 2,000 ms, TE = 30 ms, FOV = 192 mm × 192 mm, slice thickness = 3 mm, voxel size 3 × 3 × 3 mm, number of slices = 40, number of volumes: 265, matrix size: 64 × 64, no interslice gap, acquisition orientation: transverse, acquisition order: interleaved). Structural data were collected through a 3D fast spoiled gradient-echo (FSPGR) sequence (flip angle = 12°, TR = 8.2 ms, TE = 3.18 ms, slice thickness = 1 mm, voxel size 1 × 1 × 1 mm, matrix size: 256 × 256, number of slices = 136, acquisition orientation: transverse). When participants had impaired vision, we provided them with mri compatible glasses to ensure they can properly see the visual stimuli used in this experiment.

Functional image preprocessing was performed using SPM12 software (Welcome Department of Cognitive Neurology, London, UK), which builds on MATLAB. The first five volumes of fMRI scans were discarded to minimize the initial instability of magnetization. The remaining functional images from each participant were time sliced and realigned for head movements. The mean functional image was coregistered by the anatomical image and then segmented. The parameters for normalization were also derived from the segmentation step. The images were normalized to MNI space, resampled to voxels of 3 × 3 × 3 mm, and smoothed with a Gaussian kernel of 8 mm full width at half maximum (FWHM).

### fMRI Paradigm

The event-related fMRI paradigm presented four categories (happiness, disgust, fear or neutral) of modified emotional faces from the Ekman black-and-white face emotion database (Siger, 1979) and has previously been used (Yang et al., 2015). The stimuli consisted of two female faces and two male faces. We morphed different images from the database such that they depicted nine emotional intensities of the expressions of happiness, disgust and fear ranging from 10 to 100%. Each stimulus was presented for 400 ms and then completely blocked in black for 1,600 ms. The participants were asked to discriminate the target emotion observed on the depicted faces (happiness, disgust, fear, or neutral). Then, they should press the corresponding button on a response system with their index finger. This system consisted of four buttons – one for each of the presented emotions. One of the two buttons for the left hand should be pressed when either a neutral or a happy face is shown, while one of those for the right hand should be pressed when either the emotion disgust or fear is recognized. An interstimulus interval (ISI) was presented as a blank screen over a duration which was randomly chosen to range from 3 to 8 s and was used as implicit baseline in this paradigm. The entire experiment consisted of two sessions of 66 trials each, for a total of 132 trials. Each session lasted 9 min. Between the two sessions, participants had a 1-min break in the scanner. The facial stimuli depicting the neutral face was presented in 12 trials in total while each of the three emotions was presented 40 trials each. To allow the participants to get familiar with the buttons on the response system, all participants practiced this emotion recognition task on a laptop for 10 min. E-prime 2.0 software (Schneider et al., 2002) was used to control the experimental stimuli presentation and behavioral data recording, including the reaction times and accuracy of judgements.

Participants' performance during the emotion recognition task was assessed by reaction time (RT; for both hits and correct responses), sensitivities and specificities. Sensitivity is the ability of the participants to correctly identify the facial emotion. Specificity is the ability of the participants to correctly reject false emotions in the depicted faces. Sensitivity measures the relative portion of correctly identified facial emotions in a given emotional category and was calculated as follows:


$$\begin{array}{l} \frac{number\;of\;trials\;in\;which\;a\;certain\;emotion\;has\;correctly\;been\;recognized}{number\;of\;all\;trials\;depicting\;this\;certain\;emotion}. \end{array}$$

Specificity measures the relative portion of correct rejections of emotions that are not depicted in the shown faces and was calculated as follows:


$$\begin{array}{l} \frac{number\;of\;trials\;in\;which\;a\;certain\;emotion\;has\;correctly\;been\;rejected}{number\;of\;all\;trials\;depicting\;another\;emotional\;category}.^{\prime \prime } \end{array}$$

### Data Analysis

#### Analysis of Behavioral and Clinical Data

The sociodemographic, behavioral and psychopathological data were analyzed by using SPSS 20.0 (IBM Corp. Released 2011). We tested for group differences between NHS-UHR and the CG regarding sociodemographic data. By using an analysis of covariance (ANCOVA) with the covariates age and gender, we analyzed group differences in behavior during the facial emotion recognition task. To compensate for the accumulation of type I errors resulting from 12 consecutive significance tests, we used the Bonferroni correction and adjusted our initial significance threshold α = 0.05 to α_adj_ = α/12 = 0.0042.

#### Analysis of the MRI Data and Correlations Between Extracted Significant Voxelclusters and Prodromal Symptoms

The single-subject analysis and group analysis were based on a general linear model (GLM). The hemodynamic response triggered by the four conditions (neutral, happy, disgust, and fear) and the subsequent emotional recognition was modeled with a canonical hemodynamic response function (HRF). The intensities of the emotions were entered as a parametric modulator. The realignment parameters were added as multiple regressors in the single-subject GLM. The high-pass filter was set to 128 s to remove low-frequency signal drifts. Parameter estimates (b) and T-statistic images for each condition contrasting implicit baselines were calculated for each subject and extracted for the second-level analyses. The group analysis was conducted by entering the parametric estimates under each condition of each group into a flexible factorial analysis. Consistent with the hypothesis that UHR may present overactivity compared to the control group in general facial emotion processing regardless of the emotional categories, t-contrasts (NHS-UHR > CG, *p* < 0.05, FWE-corrected, *k* = 0) were conducted across all four conditions (neutral + happy + fear + disgust). Additionally, we aimed to investigate group differences between NHS-UHR and the CG regarding brain activation when they are processing each emotion category (neutral, happy, fear, disgust) compared to the aforementioned implicit baseline (ISI) using the again the aforementioned statistical procedure (*t*-test, NHS-UHR > CG, *p* < 0.05, FWE-corrected, *k* = 0). Then, we defined contrasts for each emotional category *vs*. neutral face to investigate the group differences in emotional processing. As our previous brain morphological study involving NHS-UHR reported reduced cortical complexity in the STG compared to the CG (Hou et al., 2020), we created a mask of the left STG using the SPM12 toolbox Wake Forrest University (WFU)-Pickatlas (Maldjian et al., 2003).

Next, the eigenvariates of the significant clusters in the STG were extracted using the VOI-function in SPM12, and Pearson's correlations were conducted to determine whether the significant clusters were related to the items from the SIPS measuring prodromal positive symptoms and negative symptoms.

## Results

### Sample, Sociodemographic Characteristics, and Behavioral Results

All in all, 8,404 undergraduate students were screened from two consecutive school years (*n* (first cohort) = 4,040; *n* (second cohort) = 4,364). 77.3% (3,121) PQ-16 questionnaires were returned from the students in the first school year. Of these participants, 71 reported scores above the cut-off. After the following SIPS interview, 29 participants met the criteria for being at UHR. One year later, we reassessed these NHS-UHR using the SIPS to screen the participants for the fMRI experiment. One participant was diagnosed with bipolar disorder, and nine other participants no longer presented ultra-high-risk symptoms. Only 10 participants still met the criteria for UHR status after the SIPS and participated in our fMRI experiment.

Regarding the second cohort of undergraduate students, we sent 4,364 PQ-B questionnaires and received 3,498 (80.2%) completed questionnaires. In total, 1,364 (39%) students scored above this cut-off. We again used the SIPS interview to more deeply screen the UHR status, and 16 participants (1.2%) were identified as NHS-UHR. Fourteen of these NHS-UHR from the second cohort completed the fMRI experiment, the other two dropped out of the study due to a lack of motivation to undergo an MRI scan.

In total, 51 participants completed the fMRI experiment; four participants were excluded due to excessive head movement (≥3 mm). There were no significant differences in head motion across both groups (*t*_(df = 42)_ = −0.486, *p* = 0.63). After analyzing the behavioral data, three participants were excluded because of a low response rate which was defined as a response rate <80%.

In our final fMRI analysis, 44 participants were included, including 21 NHS-UHR (15 males, 6 females; *M*(age) = 19.48, *SD* = 0.13) and 23 controls (14 males, 9 females; age = 20.39, *SD* = 0.25).

Sociodemographic information and descriptive statistics of the SIPS scores are shown in Table 1. We found no significant differences between the NHS-UHR participants and healthy controls in gender, handedness and ethnicity, i.e., belonging to the Han ethnicity [ethnical group consisting of 1.4 billion Chinese people that make up about 92% of the Chinese population (Wen et al., 2004)] vs. belonging to another Chinese ethnicity (see Table 1). However, the groups significantly differed in age (NHS-UHR: *M* = 19.48, *SD* = 0.6; CG: *M* = 20.39, *SD* = 1.2; *F* = 9.972, *p* = 0.003, *d* = 0.976).

**Table 1:** Sociodemographic characteristics and clinical symptoms in high-functioning high-risk psychosis (NHS-UHR) and control group (CG).

	**NHS-UHR**		**CG**			
	**(*****n*** **=** **21)**		**(*****n*** **=** **23)**			
	***mean***	***SD***	***mean***	***SD***	***F/χ2***	***p***
Age (year)	19.48	0.6	20.39	1.2	9.972	0.003^*^
Gender (M/F)	15/6		14/9		0.545	0.46
Handedness (R/L)	20/1		20/3		0.911	0.34
Ethnicity (Han/another Chinese ethnicity)	18/3		19/4		6.95	0.224
**SIPS**						
**Positive symptoms**	6.95	3.217				
Unusual thought Content/delusional ideas	2.00	1.000				
Suspiciousness/persecutory ideas	1.86	1.315				
Grandiosity	0.67	0.796				
Perceptual Abnormalities/hallucinations	2.29	1.617				
Disorganized Communication	0.38	0.590				
**Negative symptoms**	5.19	5.046				
Social anhedonia	1.57	1.363				
Avolition	1.05	1.465				
Expression of emotion	0.81	0.981				
Experience of emotions and self	0.86	1.062				
Ideational richness	0.33	0.658				
Occupational functioning	0.57	0.811				
**Disorganized symptoms**	2.62	2.559				
Odd behavior and appearance	0.19	0.512				
Bizarre thinking	0.57	0.870				
Trouble with focus and attention	1.57	1.287				
Personal hygiene	0.29	0.717				
**General symptoms**	3.57	3.458				
Sleep disturbance	1.05	1.359				
Dysphoric mood	1.19	0.981				
Motor disturbance	0.57	0.978				
Impaired tolerance to normal stress	0.76	1.044				
GAF	75.38	9.119				

* *p < 0.05. SIPS, semi-structured interview for prodromal symptoms; GAF, Global Assessment of Functioning Scale; CD-RISC, Connor-Davidson resilience scale*.

When comparing the GAF scores in our NHS-UHR group (*M* = 75.38; *SD* = 9.119; minimum = 64) to the mean GAF value in UHR from another student population [Fusar-Poli et al., 2010; mean (GAF) = 57] using a one-sample *t*-test, it differed significantly from the reported mean [*t*_(df = 20)_ = 9.237; *p* < 0.001]. Additionally, the GAF score in NHS-UHR group in this study was significantly lower than the mean GAF in the general population in the UK reported in another study [Hui et al., 2013; M(GAF in the general population) = 86.6, SD = 3.8; *t*_(df = 20)_ = −5.64; *p* < 0.001] and the mean GAF in the general rural Chinese population [Jia and Zhang, 2012; M(GAF) = 89.19, SD = 7.18; *t*_(df = 20)_ = −6.94; *p* < 0.001].

**Figure 1:**
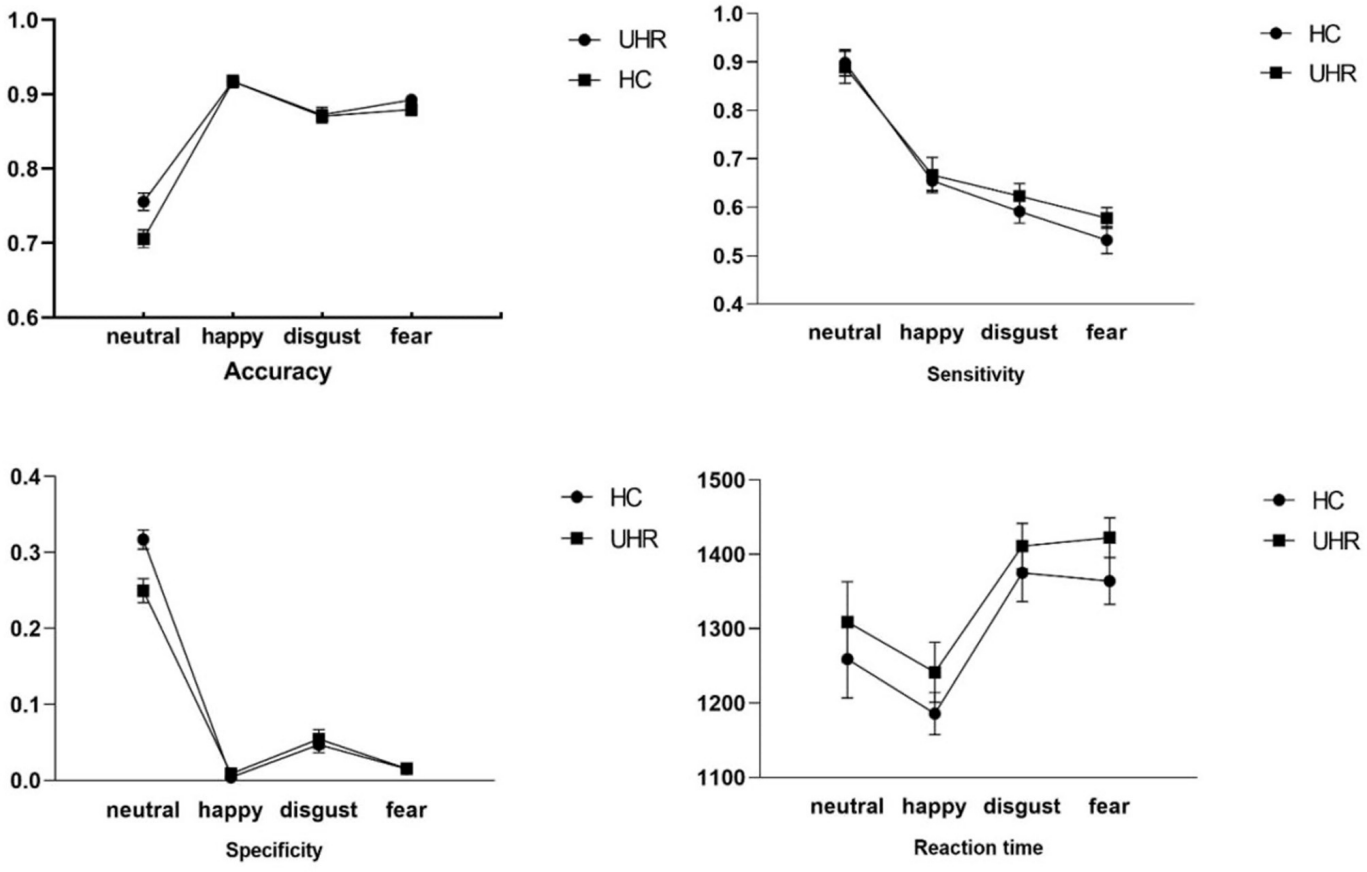
Group comparisons of accuracy, sensitivity, specificity and reaction time during happy, neutral, disgust and fear emotion recognition between UHR and control group.

In the emotion recognition task, there were no significant differences in accuracy, RT and sensitivity. Although specificity for the emotions happiness, disgust and fear did not differ among the groups (see Figure 1) NHS-UHR participants presented higher specificity when recognizing neutral emotions [*F* = 4.92, *p* = 0.03, effect size (η^2^) = 0.11]. However, this effect did not survive Bonferroni correction.

### fMRI Whole-Brain Comparisons

#### General Facial Emotion Processing (Contrasts: NHS-UHR vs. CG for Neutral + Happy + Disgust + Fear > ISI)

**Figure 2:**
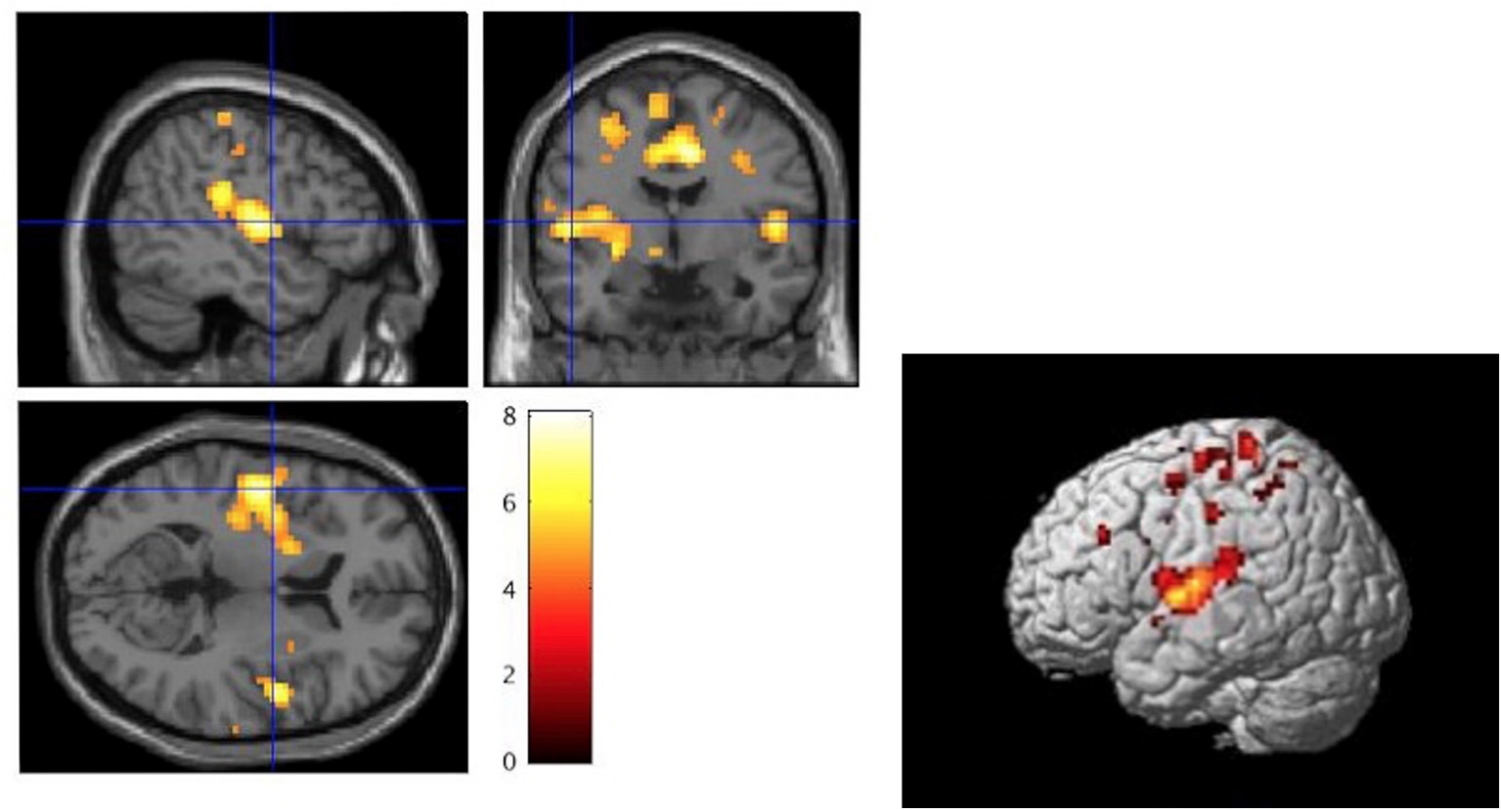
Whole brain analysis of high functioning ultra-high risk psychosis (NHS-UHR) > control group (CG) for the contrast: neutral+happy+disgust+fear during the face emotion recognition task (*p* < 0.05, FWE-corrected). The color bar represents t-values ranging from 0 to 8. The left picture displays a significant peak at 51/-1/5 (MNI Space) of a cluster ranging over the right superior temporal cortex and the left insula [Cluster labeling was executed using the automated anatomic atlas (AAL; Tzourio-Mazoyer et al., 2002)]. The left image was created with the section view provided by SPM12 and as a template the volumetric image *single_subj_T1.nii* based on the study by Holmes et al. (1998). The right image was created using render view (*render_single_subj.mat*) which is also provided by SPM12.

Compared to the CG, the NHS-UHR showed significant hyperactivities in the left and right postcentral gyrus, left Heschl's gyrus, which extended to the left insula, pallidum, inferior frontal gyrus and STG, right operculum, middle occipital lobe, left middle frontal gyrus and precentral gyrus, right superior parietal gyrus and middle cingulum (*p* < 0.05, FWE-corrected). We found no significant hypoactivities in the NHS-UHR compared to the CG. The results are presented in Table 2 and Figure 2.

**Table 2:** Whole brain analysis of functional hyperactivations in high functioning individuals at ultra-high risk for psychosis compared to control group during general facial emotion recognition.

		**MNI coordinates**						
**Cluster size k**	**Anatomic region according to the AAL**	**x**	**y**	**z**	***t***	***p***	**Extension according to the AAL**	**Cluster size**
1,011	Right postcentral gyrus	9	−4	41	7.8	<0.001	Right supramarginal gyrus	208
							Left precentral gyrus	158
							Left precentral gyrus	148
							Left midcingulate cortex	128
							Right midcingulate cortex	108
							Right supplementary motor area	103
593	Left Heschl's gyrus	−48	−10	5	7.56	<0.001	Left insula	132
							Left pallidum	108
							Left inferior frontal gyrus	90
							Left postcentral gyrus	80
74	Right rolandic operculum	51	−1	5	7.36	<0.001	Right insula	44
							Right superior temporal gyrus	24
59	Right rolandic operculum	51	−22	23	6.9	<0.001	Right rolandic operculum	29
							Right insula	17
22	Right middle occipital gyrus	30	−70	38	6.06	<0.001	Right middle occipital gyrus	15
65	Right middle frontal gyrus	42	23	32	5.9	<0.001	Right inferior frontal gyrus	51
21	Left precentral gyrus	−36	−16	65	5.73	<0.001		
18	Right superior parietal gyrus	39	−46	59	5.63	<0.001		
51	Left middle frontal gyrus	−30	−4	53	5.54	0.001	Left superior frontal gyrus	22
6	Left middle cingulate gyrus	−9	−40	41	5.45	0.001		
8	Left middle frontal gyrus	−39	29	32	5.19	0.003		
29	Left postcentral gyrus	−30	−37	68	5.18	0.003		
8	Left postcentral gyrus	−48	−25	59	5.16	0.004		

*Cluster labeling was executed using the automated anatomic atlas (AAL; Tzourio-Mazoyer et al., 2002). Coordinates are shown in MNI space*.

#### Facial Emotion Recognition vs. Baseline (Contrasts: Happy vs. ISI; Disgust vs. ISI, Fear vs. ISI and Neutral > ISI vs. ISI × NHS-UHR vs. CG)

There were no significant group differences at the set statistical thresholds.

#### Facial Emotion Recognition (Contrasts: Happy vs. Neutral; Disgust vs. Neutral and Fear vs. Neutral > ISI × NHS-UHR vs. CG)

No significant differences were found between NHS-UHR and the CG in any specific contrast (happy, disgust, and fear) after applying FWE-correction.

#### Correlations Between the Left Superior Temporal Gyrus and Positive and Negative Symptoms

Functional activity in the left STG in NHS-UHR across all emotions (first contrast, i.e., NHS-UHR vs. CG for neutral + happy + disgust + fear > ISI) was significantly correlated with one item from the SIPS measuring negative symptoms, namely the ability to experience emotions (*r* = −0.459, *p* = 0.036). We did not find any other correlations between the other negative symptoms or positive symptoms measured with the SIPS and the extracted functional activity in the STG. Functional activations in the contrasts neutral + happy + disgust + fear > ISI (NHS-UHR > CG) were also not correlated with neither positive nor negative symptoms measured with the SIPS. The aforementioned significant correlation would not survive correction for multiple testing (eleven consecutive tests).

## Discussion

In this fMRI study, we aimed to explain previous heterogenous results on facial emotion recognition in UHR by focusing on a subgroup within the broad UHR spectrum, namely NHS-UHR. We show that NHS-UHR do not show any behavioral deficits compared to a CG, but higher specificity when recognizing emotionally neutral facial expressions. NHS-UHR demonstrated hyperactivities in the superior temporal and left Heschl's gyrus as well as in the somatosensory, insular and midcingulate cortex during facial emotion recognition. Functional activity in the STG in NHS-UHR was negatively correlated with the severity of negative symptoms in NHS-UHR.

Our first main finding is that, at a behavioral level, NHS-UHR perform very similar to healthy controls when recognizing facial emotions. Empirical evidence from studies investigating behavioral performance during facial emotion recognition in UHR has been mixed (Lee et al., 2015; van Donkersgoed et al., 2015). In a case-control study involving high-functioning schizophrenia patients and healthy subjects no behavioral group differences were found during facial emotion perception (Karpouzian et al., 2017). Another study (Palmer et al., 2018) using an affective face matching task (Hariri et al., 2000) in order to investigate one high-functioning schizophrenia patient showed that this patient performed better than other non-high-functioning patients with schizophrenia and similar to a non-psychiatric control group. It is important to emphasize that deriving conclusions from a study investigating only one subject, thus not having a basis for generalization, remains highly speculative. However, summarizing all these study results, we speculate that within the psychosis spectrum, a phenotype that exhibits high community functioning may perform better in facial emotion perception (and potentially shows better neuropsychological functioning in other cognitive domains) than other UHR or schizophrenia patients. Consistent with this notion, a previous study found that real-life functioning in UHR is strongly related to the processing speed of social cognitive information (Glenthøj, 2018) and discussed these findings from the perspective of a disruption of the more automatic/effortless processes in normal emotion recognition in UHR. In line with this interpretation, our study results show no differences in reaction times across groups.

We found bilateral hyperactivations in the STG. This finding is in line with a study on youth with psychosis spectrum symptoms investigating brain activity during an emotional processing task (Wolf et al., 2015). The STG is part of the essential face processing network and contributes to the integration of multisensory emotional stimuli in consideration of experience and social knowledge (Terasawa et al., 2013; Pehrs et al., 2014). It plays a key role in recognizing nonverbal social cues and in inferring the intentions of others (Ochsner, 2008). Multiple studies have shown that the morphology of the STG is multimodally changed in UHR, including gray matter volumes (Ding et al., 2019), white matter integrity (Vijayakumar et al., 2016) and cortical folding (Hou et al., 2020). It has been suggested that clinical high risk for psychosis may result from dysfunctions in medial temporal (and frontotemporal) regions due to increased excitatory neurotransmission which could eventually lead to dopamine dysregulation, aberrant salience and delusional ideation (Allen et al., 2019).

In our exploratory analysis, functional activity in the left STG was significantly negatively correlated with deficits in experiencing emotions in general when not correcting for multiple testing. While functional activities in these regions were previously interpreted as a potential pathological feature (Kozhuharova et al., 2020), this finding could also mean that they might represent a compensatory process that supports the preservation of facial emotion recognition by reducing deficits in experiencing emotion in NHS-UHR. There were no other associations between the functional activity in the left STG and positive symptoms in NHS-UHR, indicating that this anatomical region may play a specific role in the ability of UHR to experience emotions. These findings may inspire early interventions for individuals at high risk for psychosis that target the left STG. It has already been shown that different forms of psychotherapy are able to affect brain activation in patients with schizophrenia (Kumari et al., 2011,Barsaglini et al., 2014).

Other hyperactivations we found in this study that involve brain areas that play an important role in facial emotion recognition include the insula, the cingulate cortex and the somatoform cortex. The insula is a subcortical brain region which contributes to processing emotional information (Fusar-Poli et al., 2009; Shepherd et al., 2012) and to the representation of current social contexts and also to the integration of affective and cognitive processes (Berntson et al., 2011). Its function helps making social decisions in uncertain situations considering social emotional information (Lamm and Singer, 2010). Cingulate cortices are involved in integrating emotional information to motivate behavior, conditioned emotional learning (Bush et al., 2000; Phan et al., 2002). The supramarginal gyrus and the operculum are parts of the secondary somatosensory cortex. It has been shown that the perception of facial emotions might depend on the somatosensory representation relating to this emotion (Hoekert et al., 2008). While the aforementioned brain areas also showed hyperactivations during emotion processing in some other studies on individuals at high risk for psychosis [insula (Modinos et al., 2015), cingulate cortex tasks (Derntl et al., 2015; van der Velde et al., 2015), somatosensory cortices (Gee et al., 2012; Kozhuharova et al., 2020)] many other studies were not able to replicate these findings (Kozhuharova et al., 2020; Lukow et al., 2021). These inconsistencies might in part be a result from heterogeneity of samples included in these studies. For example, it is unclear which portions of the samples will transition to frank psychosis. Additionally, UHR usually have another axis I disorder (Fusar-Poli et al., 2012b; Shi et al., 2017) which are associated with functional brain activity changes during facial emotion processing (Bourke et al., 2010), often use psychopharmacological medication that can affect brain activity (Paulus et al., 2005; Murphy et al., 2009) and, due to the current conceptualization of being at high risk for psychosis, there are different diagnostic criteria that can be fulfilled that can also be concurrent in order to be considered an UHR. This work can help to disentangle these effects by including a specific homogeneous sample that was also carefully screened to ensure absence of other mental disorders and psychopharmacological medication in all participants.

One major focus of this study was the investigation of behavioral and neural correlates of neutral facial emotion recognition. While there was a statistically significant effect suggesting that NHS-UHR show higher specificity compared to CG when recognizing neutral facial emotions (*p* = 0.03; note that this effect did not survive Bonferroni-correction), we did not find any differences in brain activation between these two groups (in contrast to other sudies on UHR: Seiferth et al., 2008; Modinos et al., 2015; van der Velde et al., 2015). There is empirical evidence that both UHR (van Rijn et al., 2011) and schizophrenia patients (Kohler et al., 2003) label neutral facial expressions as negatively valanced compared to healthy controls. According to psychological models of psychosis, these misinterpretations of neutral emotions as threatening could impact on the development of positive psychotic symptoms (Garety et al., 2007). Another study (Allott et al., 2014) suggests that neutral facial emotion recognition abilities could be used as a prognostic marker for transition into psychosis since converters mislabelled neutral faces as fearful after controlling for symptoms, functioning and age. In the context of these findings, the results from the present study might stimulate further research on whether the correct identification of neutral facial emotion acts as a protective factor that preserves functioning levels and, potentially, prevents transition into frank psychosis.

## Limitations

There are some limitations that should be considered. Despite the first screening of a large cohort of University students, our sample size remains rather low, which limits the statistical power of our analyses and our ability to detect potential behavioral or neurofunctional differences between the NHS-UHR and the CG. Using G^*^Power (version 3.1.9.6; Faul et al., 2007), we computed a sensitivity analysis (i.e., the population effect size is calculated as a function of α, 1-β and *N*) in order to determine the minimal detectable effect (MDE) when conducting a two sample *t*-test with our data, i.e., what level of effect we can find given our sample size (*n*_1_= 21 and *n*_2_= 23) and a set type I error probability of α = 0.05 and a set power (1- *p*(β) of 0.8. The estimated critical *t*-value required for detecting significant effects with our sample size is *t* = ±2.02 with a minimum effect size *d* = 0.87. Next, we interpreted a null result as empirical evidence for our first hypothesis which could be the mistaken acceptance of an actually false null hypothesis (type II error) e.g., due to the lack of statistical power which would have been necessary in order to detect existent group differences. Also, since we were only investigating a student population from an elite University, the generalisability of our results is limited. In addition, the use of such a population, despite complete anonymisation of the data, raises the question of whether the participants answered less honestly, since their data were analyzed by researchers from the University at which they themselves study and stigma-related stress. Since functioning is a complex, multi-dimensional construct, future studies should investigate which aspects primarily shape group differences as well as similarities reported in this study. Future fMRI studies on this topic should explore general face processing by conducting a gender discrimination task to isolate the neuroactivities involved in face emotion processing on the one hand and emotion processing on the other.

## Conclusion

Our results demonstrate that the ability to recognize facial emotions is preserved in NHS-UHR. This perseveration was accompanied by hyperactivities in the premotor cortex, somatosensory cortex, the temporoparietal-occipital visual network, the insula and the STG which play an important role in facial emotion recognition. Our study may inspire future early intervention and provide loci for treatment using neural stimulation.

## Data Availability Statement

The original contributions presented in the study are included in the article/supplementary material, further inquiries can be directed to the corresponding authors.

## Ethics Statement

The studies involving human participants were reviewed and approved by the Ethics Committee of the Institutional Review Board of Tongji University. The patients/participants provided their written informed consent to participate in this study.

## Author Contributions

JH: data acquisition, data analysis, original draft preparation, and manuscript writing. SS: data analysis, interpretation, manuscript writing, and manuscript revision. XZ: supervision of experimental design, data acquisition, and manuscript revision. JW and JC: data analysis and manuscript revision. ZM and AQ: data acquisition and manuscript editing. ZL: supervision of experimental design and data acquisition. TK: supervision of data analysis and manuscript revision. YY: data analysis, interpretation, manuscript writing, and revision. JS: funding acquisition, supervision of the study design and manuscript revision. All authors contributed to the article and approved the submitted version.

## Funding

This study was supported by the National Natural Science Foundation of China (Grant No.: 31600892), Fundamental Research Funds for the Central Universities of Tongji University (Grant No.: 22120180542), the Outstanding Clinical Discipline Project of Shanghai Pudong (Grant No.: PWYgy2018-10), and Science Project of Shanghai Municipal Health Commission (grant number: 202040030).

## Conflict of Interest

TK received unrestricted educational grants from Servier, Janssen, Recordati, Aristo, Otsuka, neuraxpharm. The remaining authors declare that the research was conducted in the absence of any commercial or financial relationships that could be construed as a potential conflict of interest.

## Publisher's Note

All claims expressed in this article are solely those of the authors and do not necessarily represent those of their affiliated organizations, or those of the publisher, the editors and the reviewers. Any product that may be evaluated in this article, or claim that may be made by its manufacturer, is not guaranteed or endorsed by the publisher.

## References

[B1] Allen P. Moore H. Corcoran C. M. Gilleen J. Kozhuharova P. Reichenberg A.. (2019). Emerging temporal lobe dysfunction in people at clinical high risk for psychosis. Front. Psychiatry. 10, 298. 10.3389/fpsyt.2019.00298 31133894 PMC6526750

[B2] Allott K. A. Schäfer M. R. Thompson A. Nelson B. Bendall S. Bartholomeusz C. F.. (2014). Emotion recognition as a predictor of transition to a psychotic disorder in ultra-high risk participants. Schizophr. Res. 153, 25–31. 10.1016/j.schres.2014.01.037 24552619

[B3] Amminger G. P. Schäfer M. R. Papageorgiou K. Klier C. M. Schlögelhofer M. Mossaheb N.. (2012). Emotion recognition in individuals at clinical high-risk for schizophrenia. Schizophr. Bull. 38, 1030–1039. 10.1093/schbul/sbr015 21422108 PMC3446213

[B4] Ayoub I. A. Andrade J. C. Serpa M. H. Alves T. M. Hortêncio L. Freitas E. L.. (2020). Relationship between symptomatic dimensions and global functioning of non–help-seeking individuals at risk for psychosis. J. Nerv. Ment. Dis. 208, 953–957. 10.1097/NMD.0000000000001237 32925694

[B5] Barkl S. J. Lah S. Harris A. W. Williams L. M. (2014). Facial emotion identification in early-onset and first-episode psychosis: a systematic review with meta-analysis. Schizophr. Res. 159, 62–69. 10.1016/j.schres.2014.07.049 25178803

[B6] Barsaglini A. Sartori G. Benetti S. Pettersson-Yeo W. Mechelli A. (2014). The effects of psychotherapy on brain function: a systematic and critical review. Prog. Neurobiol. 114, 1–14. 10.1016/j.pneurobio.2013.10.006 24189360

[B7] Berntson G. G. Norman G. J. Bechara A. Bruss J. Tranel D. Cacioppo J. T. (2011). The insula and evaluative processes. Psychol. Sci. 22, 80–86. 10.1177/0956797610391097 21148459 PMC3261800

[B8] Bourke C. Douglas K. Porter R. (2010). Processing of facial emotion expression in major depression: a review. Aust. N. Z. J. Psychiatry. 44, 681–696. 10.3109/00048674.2010.496359 20636189

[B9] Bush G. Luu P. Posner M. I. (2000). Cognitive and emotional influences in anterior cingulate cortex. Trends Cogn. Sci. 4, 215–222. 10.1016/S1364-6613(00)01483-2 10827444

[B10] Chen F. Wang L. Heeramun-Aubeeluck A. Wang J. Shi J. Yuan J.. (2014). Identification and characterization of college students with attenuated psychosis syndrome in China. Psychiatry Res. 216, 346–350. 10.1016/j.psychres.2014.01.051 24636247

[B11] Craig A. D. (2009). How do you feel–now? The anterior insula and human awareness. Nat. Rev. Neurosci. 10, 59–70. 10.1038/nrn2555 19096369

[B12] Dalili M. N. Penton-Voak I. S. Harmer C. J. Munaf,ò M. R. (2015). Meta-analysis of emotion recognition deficits in major depressive disorder. Psychol. Med. 45, 1135–1144. 10.1017/S0033291714002591 25395075 PMC4712476

[B13] Derntl B. Michel T. M. Prempeh P. Backes V. Finkelmeyer A. Schneider F.. (2015). Empathy in individuals clinically at risk for psychosis: brain and behaviour. Br. J. Psychiatry. 207, 407–413. 10.1192/bjp.bp.114.159004 26294367

[B14] Di Ieva A. Grizzi F. Jelinek H. Pellionisz A. J. Losa G. A. (2013). Fractals in the neurosciences, part I: general principles and basic neurosciences. Neuroscientist. 20, 403–417. 10.1177/1073858413513927 24362815

[B15] Ding Y. Ou Y. Pan P. Shan X. Chen J. Liu F.. (2019). Brain structural abnormalities as potential markers for detecting individuals with ultra-high risk for psychosis: a systematic review and meta-analysis. Schizophr. Res. 209, 22–31. 10.1016/j.schres.2019.05.015 31104914

[B16] Ekman P. (1993). Facial expression and emotion. Am. Psychol. 48, 384–392. 10.1037/0003-066X.48.4.384 8512154

[B17] Endicott J. Spitzer R. L. Fleiss J. L. Cohen J. (1976). The global assessment scale: a procedure for measuring overall severity of psychiatric disturbance. Arch. Gen. Psychiatry. 33, 766–771. 10.1001/archpsyc.1976.01770060086012 938196

[B18] Falkenberg I. Valmaggia L. Byrnes M. Frascarelli M. Jones C. Rocchetti M.. (2015). Why are help-seeking subjects at ultra-high risk for psychosis help-seeking? Psychiatry Res. 228, 808–815. 10.1016/j.psychres.2015.05.018 26071897

[B19] Faul F. Erdfelder E. Lang A.-G. Buchner A. (2007). G^*^Power 3: A flexible statistical power analysis program for the social, behavioral, and biomedical sciences. Behav. Res. Methods. 39, 175–191. 10.3758/BF03193146 17695343

[B20] Fusar-Poli P. (2017). Why ultra high risk criteria for psychosis prediction do not work well outside clinical samples and what to do about it. World Psychiatry. 16, 212–213. 10.1002/wps.20405 28498578 PMC5428173

[B21] Fusar-Poli P. Borgwardt S. Bechdolf A. Addington J. Riecher-Rössler A. Schultze-Lutter F.. (2013). The psychosis high-risk state: a comprehensive state-of-the-art review. JAMA Psychiatry. 70, 107–120. 10.1001/jamapsychiatry.2013.269 23165428 PMC4356506

[B22] Fusar-Poli P. Byrne M. Valmaggia L. Day F. Tabraham P. Johns L.. (2010). Social dysfunction predicts two years clinical outcome in people at ultra high risk for psychosis. J. Psychiatr. Res. 44, 294–301. 10.1016/j.jpsychires.2009.08.016 19836755

[B23] Fusar-Poli P. Cappucciati M. Borgwardt S. Woods S. W. Addington J. Nelson B.. (2015a). Heterogeneity of psychosis risk within individuals at clinical high risk: a meta-analytical stratification. JAMA Psychiatry. 1–8, 113–120. 10.1001/jamapsychiatry.2015.2324 26719911

[B24] Fusar-Poli P. Cappucciati M. Rutigliano G. Lee T. Y. Beverly Q. Bonoldi I.. (2016). Towards a standard psychometric diagnostic interview for subjects at ultra high risk of psychosis: CAARMS versus SIPS. Psychiatry J. 2016, 1–11. 10.1155/2016/7146341 27314005 PMC4904115

[B25] Fusar-Poli P. Deste G. Smieskova R. Barlati S. Yung A. R. Howes O.. (2012a). Cognitive functioning in prodromal psychosis: a meta-analysis. Arch. Gen. Psychiatry. 69, 562. 10.1001/archgenpsychiatry.2011.1592 22664547

[B26] Fusar-Poli P. Nelson B. Valmaggia L. Yung A. R. McGuire P. K. (2012b). Comorbid Depressive and Anxiety Disorders in 509 Individuals With an At-Risk Mental State: Impact on Psychopathology and Transition to Psychosis. Schizophr. Bull. 40, 120–131. 10.1093/schbul/sbs136 23180756 PMC3885287

[B27] Fusar-Poli P. Placentino A. Carletti F. Landi P. Allen P. Surguladze S.. (2009). Functional atlas of emotional faces processing: A voxel-based meta-analysis of 105 functional magnetic resonance imaging studies. J. Psychiatry. Neurosci. 34, 418–432. 19949718 PMC2783433

[B28] Fusar-Poli P. Rocchetti M. Sardella A. Avila A. Brandizzi M. Caverzasi E.. (2015b). Disorder, not just state of risk: Meta-analysis of functioning and quality of life in people at high risk of psychosis. Br. J. Psychiatry. 207, 198–206. 10.1192/bjp.bp.114.157115 26329563

[B29] Fusar-Poli P. Tantardini M. De Simone S. Ramella-Cravaro V. Oliver D. Kingdon J.. (2017). Deconstructing vulnerability for psychosis: meta-analysis of environmental risk factors for psychosis in subjects at ultra high-risk. Eur. Psychiatry. 40, 65–75. 10.1016/j.eurpsy.2016.09.003 27992836

[B30] Gabay A. S. Kempton M. J. Mehta M. A. (2015). Facial affect processing deficits in schizophrenia: a meta-analysis of antipsychotic treatment effects. J. Psychopharmacol. 29, 224–229. 10.1177/0269881114560184 25492885 PMC4361469

[B31] Garety P. A. Bebbington P. Fowler D. Freeman D. Kuipers E. (2007). Implications for neurobiological research of cognitive models of psychosis: a theoretical paper. Psychol. Med. 37, 1377–1391. 10.1017/S003329170700013X 17335638

[B32] Gee D. G. Karlsgodt K. H. van Erp T. G. Bearden C. E. Lieberman M. D. Belger A.. (2012). Altered age-related trajectories of amygdala-prefrontal circuitry in adolescents at clinical high risk for psychosis: a preliminary study. Schizophr. Res. 134, 1–9. 10.1016/j.schres.2011.10.005 22056201 PMC3245800

[B33] Glenthøj L. (2018). *Emotion recognition latency, but not accuracy*, relates to real life functioning in individuals at ultra-high risk for psychosis. Schizophr. Res. 197–202. 10.1016/j.schres.2018.12.038 30595441

[B34] Hariri A. R. Bookheimer S. Y. Mazziotta J. C. (2000). Modulating emotional responses: effects of a neocortical network on the limbic system. NeuroReport. 11:43–48. 10.1097/00001756-200001170-00009 10683827

[B35] Harmer C. J. Dawson G. R. Dourish C. T. Favaron E. Parsons E. Fiore M.. (2013). Combined NK1 antagonism and serotonin reuptake inhibition: effects on emotional processing in humans. J. Psychopharmacol. 27, 435–443. 10.1177/0269881112472558 23407644

[B36] Harmer C. J. de Bodinat C. Dawson G. R. Dourish C. T. Waldenmaier L. Adams S.. (2011). Agomelatine facilitates positive versus negative affective processing in healthy volunteer models. J. Psychopharmacol. 25, 1159–1167. 10.1177/0269881110376689 20660010

[B37] Hoekert M. Bais L. Kahn R. S. Aleman A. (2008). Time course of the involvement of the right anterior superior temporal gyrus and the right fronto-parietal operculum in emotional prosody perception. PLoS ONE. 3, e2244. 10.1371/journal.pone.0002244 18493307 PMC2373925

[B38] Holmes C. J. Hoge R. Collins L. Woods R. Toga A. W. Evans A. C. (1998). Enhancement of MR images using registration for signal averaging. J. Comput. Assist. Tomog. 22, 324–333. 10.1097/00004728-199803000-00032 9530404

[B39] Hou J. Schmitt S. Meller T. Falkenberg I. Chen J. Wang J.. (2020). Cortical complexity in people at ultra-high-risk for psychosis moderated by childhood trauma. Front. Psychiatry. 11, 1236. 10.3389/fpsyt.2020.594466 33244301 PMC7685197

[B40] Hui C. Morcillo C. Russo D. A. Stochl J. Shelley G. F. Painter M.. (2013). Psychiatric morbidity, functioning and quality of life in young people at clinical high risk for psychosis. Schizophr. Res. 148, 175–180. 10.1016/j.schres.2013.05.026 23773297 PMC3744805

[B41] Ising H. K. Veling W. Loewy R. L. Rietveld M. W. Rietdijk J. Dragt S.. (2012). The Validity of the 16-Item version of the prodromal questionnaire (PQ-16) to screen for ultra high risk of developing psychosis in the general help-seeking population. Schizophr. Bull. 38, 1288–1296. 10.1093/schbul/sbs068 22516147 PMC3713086

[B42] Jáni M. Kašpárek T. (2018). Emotion recognition and theory of mind in schizophrenia: a meta-analysis of neuroimaging studies. World J. Biol. Psychiatry. 19, S86–S96. 10.1080/15622975.2017.1324176 28449613

[B43] Jia C.-X. Zhang J. (2012). Global functioning and suicide among chinese rural population aged 15–34 years: a psychological autopsy case-control study^*^. J. Forensic Sci. 57, 391–397. 10.1111/j.1556-4029.2011.01978.x 22150171

[B44] Johnston P. J. Stojanov W. Devir H. Schall U. (2005). Functional MRI of facial emotion recognition deficits in schizophrenia and their electrophysiological correlates. Eur. J. Neurosci. 22, 1221–1232. 10.1111/j.1460-9568.2005.04294.x 16176365

[B45] Karpouzian T. M. Schroeder M. P. Abram S. V. Wanar H. Alden E. C. Eack S. M.. (2017). Neural correlates of preserved facial affect perception in high functioning schizophrenia. Psychiatry Res. Neuroimaging. 266, 83–85. 10.1016/j.pscychresns.2017.06.002 28624640 PMC10725252

[B46] Kohler C. G. Turner T. H. Bilker W. B. Brensinger C. M. Siegel S. J. Kanes S. J.. (2003). Facial emotion recognition in schizophrenia: intensity effects and error pattern. Am. J. Psychiatry. 160, 1768–1774. 10.1176/appi.ajp.160.10.1768 14514489

[B47] Kozhuharova P. Saviola F. Ettinger U. Allen P. (2020). Neural correlates of social cognition in populations at risk of psychosis: a systematic review. Neurosci. Biobehav. Rev. 108, 94–111. 10.1016/j.neubiorev.2019.10.010 31730786

[B48] Kumari V. Fannon D. Peters E. R. ffytche D. H. Sumich A. L. Premkumar P.. (2011). Neural changes following cognitive behaviour therapy for psychosis: a longitudinal study. Brain. 134, 2396–2407. 10.1093/brain/awr154 21772062 PMC3155705

[B49] Lamm C. Singer T. (2010). The role of anterior insular cortex in social emotions. Brain Struct Funct. 214, 579–591. 10.1007/s00429-010-0251-3 20428887

[B50] Lee T. Y. Hong S. B. Shin N. Y. Kwon J. S. (2015). Social cognitive functioning in prodromal psychosis: a meta-analysis. Schizophr. Res. 164, 28–34. 10.1016/j.schres.2015.02.008 25749019

[B51] Loewy R. L. Pearson R. Vinogradov S. Bearden C. E. Cannon T. D. (2011). Psychosis risk screening with the prodromal questionnaire–brief version (PQ-B). Schizophr. Res. 129, 42–46. 10.1016/j.schres.2011.03.029 21511440 PMC3113633

[B52] Lukow P. B. Kiemes A. Kempton M. J. Turkheimer F. E. McGuire P. Modinos G. (2021). Neural correlates of emotional processing in psychosis risk and onset–a systematic review and meta-analysis of fMRI studies. Neurosci. Biobehav. Rev. 128, 780–788. 10.1016/j.neubiorev.2021.03.010 33722617 PMC8345001

[B53] Maldjian J. A. Laurienti P. J. Kraft R. A. Burdette J. H. (2003). An automated method for neuroanatomic and cytoarchitectonic atlas-based interrogation of fMRI data sets. NeuroImage. 19, 1233–1239. 10.1016/S1053-8119(03)00169-1 12880848

[B54] Martin D. Croft J. Pitt A. Strelchuk D. Sullivan S. Zammit S. (2020). Systematic review and meta-analysis of the relationship between genetic risk for schizophrenia and facial emotion recognition. Schizophr. Res. 218, 7–13. 10.1016/j.schres.2019.12.031 31932173

[B55] Martin F. Baudouin J. Y. Tiberghien G. Franck N. (2005). Processing emotional expression and facial identity in schizophrenia. Psychiatry Res. 134, 43–53. 10.1016/j.psychres.2003.12.031 15808289

[B56] McGlashan T. Miller T. Woods S. Rosen J. Hoffman R. Davidson L. (2001). Structured Interview for Prodromal Syndromes. New Haven, CT: PRIME Research Clinic, Yale School of Medicine.

[B57] Miller T. J. Mcglashan T. H. Rosen J. L. Cadenhead K. Ventura J. Mcfarlane W.. (2003). Prodromal assessment with the structured interview for prodromal syndromes and the scale of prodromal symptoms: predictive validity, interrater reliability, and training to reliability. Schizophr. Bull. 29, 703–715. 10.1093/oxfordjournals.schbul.a007040 14989408

[B58] Modinos G. Tseng H. H. Falkenberg I. Samson C. Mcguire P. Allen P. (2015). Neural correlates of aberrant emotional salience predict psychotic symptoms and global functioning in high-risk and first-episode psychosis. Soc. Cogn. Affect. Neurosci. 10, 1429. 10.1093/scan/nsv035 25809400 PMC4590543

[B59] Murphy S. E. Norbury R. O'Sullivan U. Cowen P. J. Harmer C. J. (2009). Effect of a single dose of citalopram on amygdala response to emotional faces. Br. J. Psychiatry. 194, 535–540. 10.1192/bjp.bp.108.056093 19478294 PMC2802527

[B60] Ochsner K. N. (2008). The social-emotional processing stream: five core constructs and their translational potential for schizophrenia and beyond. Biol. Psychiatry. 64, 48–61. 10.1016/j.biopsych.2008.04.024 18549876 PMC2453243

[B61] Palmer B. W. Moore R. C. Eyler L. T. Pinto L. L. Saks E. R. Jeste D. V. (2018). Avoidance of accelerated aging in schizophrenia?: clinical and biological characterization of an exceptionally high functioning individual. Schizophr. Res. 196, 45–52. 10.1016/j.schres.2017.07.052 28801195 PMC6424115

[B62] Paulus M. P. Feinstein J. S. Castillo G. Simmons A. N. Stein M. B. (2005). Dose-Dependent decrease of activation in bilateral amygdala and insula by lorazepam during emotion processing. Arch. Gen. Psychiatry. 62, 282–288. 10.1001/archpsyc.62.3.282 15753241

[B63] Pehrs C. Deserno L. Bakels J.-H. Schlochtermeier L. H. Kappelhoff H. Jacobs A. M.. (2014). How music alters a kiss: superior temporal gyrus controls fusiform–amygdalar effective connectivity. Soc. Cogn. Affect. Neurosci. 9, 1770–1778. 10.1093/scan/nst169 24298171 PMC4221214

[B64] Phan K. L. Wager T. Taylor S. F. Liberzon I. (2002). Functional neuroanatomy of emotion: a meta-analysis of emotion activation studies in PET and fMRI. NeuroImage 16, 331–348. 10.1006/nimg.2002.1087 12030820

[B65] Phillips M. L. Drevets W. C. Rauch S. L. Lane R. (2003). Neurobiology of emotion perception I: the neural basis of normal emotion perception. Biol. Psychiatry. 54, 504–514. 10.1016/S0006-3223(03)00168-9 12946879

[B66] Rietdijk J. Ising H. K. Dragt S. Klaassen R. Nieman D. Wunderink L.. (2013). Depression and social anxiety in help-seeking patients with an ultra-high risk for developing psychosis. Psychiatry Res. 209, 309–313. 10.1016/j.psychres.2013.01.012 23433870

[B67] Sachs G. Steger-Wuchse D. Kryspin-Exner I. Gur R. C. Katschnig H. (2004). Facial recognition deficits and cognition in schizophrenia. Schizophr. Res. 68, 27–35. 10.1016/S0920-9964(03)00131-2 15037337

[B68] Savill M. D'Ambrosio J. Cannon T. D. Loewy R. L. (2018). Psychosis risk screening in different populations using the prodromal questionnaire: a systematic review. Early Interv. Psychiatry. 12, 3–14. 10.1111/eip.12446 28782283 PMC5812357

[B69] Schimmelmann B. G. Michel C. Martz-Irngartinger A. Linder C. Schultze-Lutter F. (2015). Age matters in the prevalence and clinical significance of ultra-high-risk for psychosis symptoms and criteria in the general population: findings from the BEAR and BEARS-kid studies. World Psychiatry. 14, 189–197. 10.1002/wps.20216 26043337 PMC4471976

[B70] Schneider W. Eschman A. Zuccolotto A. (2002). E-Prime Reference Guide. Pittsburg: Psychology Software Tools Inc.

[B71] Schultze-Lutter F. Michel C. Ruhrmann S. Schimmelmann B. G. (2018). Prevalence and clinical relevance of interview-assessed psychosis-risk symptoms in the young adult community. Psychol. Med. 48, 1167–1178. 10.1017/S0033291717002586 28889802 PMC6088777

[B72] Schultze-Lutter F. Michel C. Schmidt S. J. Schimmelmann B. G. Maric N. P. Salokangas R. K. R.. (2015). EPA guidance on the early detection of clinical high risk states of psychoses. Eur. Psychiatry 30, 405–416. 10.1016/j.eurpsy.2015.01.010 25735810

[B73] Seiferth N. Y. Pauly K. Habel U. Kellermann T. Shah N. J. Ruhrmann S.. (2008). Increased neural response related to neutral faces in individuals at risk for psychosis. NeuroImage. 40, 289–297. 10.1016/j.neuroimage.2007.11.020 18187342

[B74] Shepherd A. M. Matheson S. L. Laurens K. R. Carr V. J. Green M. J. (2012). Systematic meta-analysis of insula volume in schizophrenia. Biol. Psychiatry. 72, 775–784. 10.1016/j.biopsych.2012.04.020 22621997

[B75] Shi J. Wang L. Yao Y. Su N. Zhan C. Mao Z.. (2017). Comorbid mental disorders and 6-month symptomatic and functioning outcomes in Chinese University students at clinical high risk for psychosis. Front. Psychiatry. 8, 209. 10.3389/fpsyt.2017.00209 29109690 PMC5660058

[B76] Siger L. P. (1979). Unmasking The Face, A Guide To Recognizing Emotions From Facial Clues - Ekman,P, Friesen,WV. Am. Ann. Deaf. 124, 344–345.

[B77] Sliwinska M. W. Pitcher D. (2018). TMS demonstrates that both right and left superior temporal sulci are important for facial expression recognition. NeuroImage 183, 394–400. 10.1016/j.neuroimage.2018.08.025 30130641

[B78] Su N. Wang L. Shi J. Zhao X. (2015). Reliability and validity of Prodromal Questionnaire (PQ-16) in assessing psychosis-risk of college students. Journal of Tongji University. 36, 123–127.

[B79] Taylor S. F. Kang J. Brege I. S. Tso I. F. Hosanagar A. Johnson T. D. (2012). Meta-analysis of functional neuroimaging studies of emotion perception and experience in schizophrenia. Bio. Psychiatry. 71, 136–145. 10.1016/j.biopsych.2011.09.007 21993193 PMC3237865

[B80] Terasawa Y. Fukushima H. Umeda S. (2013). How does interoceptive awareness interact with the subjective experience of emotion? An fMRI study. Hum. Brain Mapp. 34, 598–612. 10.1002/hbm.21458 22102377 PMC6870042

[B81] Thompson E. Kline E. Ellman L. M. Mittal V. Reeves G. M. Schiffman J. (2015). Emotional and behavioral symptomatology reported by help-seeking youth at clinical high-risk for psychosis. Schizophr. Res. 162, 79–85. 10.1016/j.schres.2015.01.023 25638728

[B82] Tzourio-Mazoyer N. Landeau B. Papathanassiou D. Crivello F. Etard O. Delcroix N.. (2002). Automated anatomical labeling of activations in SPM using a macroscopic anatomical parcellation of the MNI MRI single-subject brain. NeuroImage 15, 273–289. 10.1006/nimg.2001.0978 11771995

[B83] van der Velde J. Opmeer E. M. Liemburg E. J. Bruggeman R. Nieboer R. Wunderink L.. (2015). Lower prefrontal activation during emotion regulation in subjects at ultrahigh risk for psychosis: an fMRI-study. NPJ Schizophrenia 1, 15026. 10.1038/npjschz.2015.26 27336036 PMC4849453

[B84] van Donkersgoed R. J. M. Wunderink L. Nieboer R. Aleman A. Pijnenborg G. H. M. (2015). Social cognition in individuals at ultra-high risk for psychosis: a meta-analysis. PLoS ONE. 10, e0141075. 10.1371/journal.pone.0141075 26510175 PMC4624797

[B85] van Rijn S. Aleman A. de Sonneville L. Sprong M. Ziermans T. Schothorst P.. (2011). Misattribution of facial expressions of emotion in adolescents at increased risk of psychosis: the role of inhibitory control. Psychol. Med. 41, 499–508. 10.1017/S0033291710000929 20507669

[B86] Vijayakumar N. Bartholomeusz C. Whitford T. Hermens D. F. Nelson B. Rice S.. (2016). White matter integrity in individuals at ultra-high risk for psychosis: a systematic review and discussion of the role of polyunsaturated fatty acids. BMC Psychiatry. 16, 287. 10.1186/s12888-016-0932-4 27515430 PMC4982267

[B87] Wen B. Li H. Lu D. Song X. Zhang F. He Y.. (2004). Genetic evidence supports demic diffusion of Han culture. Nature. 431, 302–305. 10.1038/nature02878 15372031

[B88] Wolf D. H. Satterthwaite T. D. Calkins M. E. Ruparel K. Elliott M. A. Hopson R. D.. (2015). Functional neuroimaging abnormalities in youth with psychosis spectrum symptoms. JAMA Psychiatry. 72, 456–465. 10.1001/jamapsychiatry.2014.3169 25785510 PMC4581844

[B89] Xu L. Zhang T. Zheng L. Li H. Tang Y. Luo X.. (2016). Psychometric properties of prodromal questionnaire-brief version among Chinese help-seeking individuals. PLoS ONE. 11, e0148935. 10.1371/journal.pone.0148935 26859774 PMC4747512

[B90] Yang C. Zhang T. Li Z. Heeramun-Aubeeluck A. Liu N. Huang N.. (2015). The relationship between facial emotion recognition and executive functions in first-episode patients with schizophrenia and their siblings. BMC Psychiatry. 15, 241. 10.1186/s12888-015-0618-3 26449211 PMC4599651

[B91] Yung A. R. Nelson B. (2013). The ultra-high risk concept—a review. Can. J. Psychiatry. 58, 5–12. 10.1177/070674371305800103 23327750

[B92] Yung A. R. Nelson B. Stanford C. Simmons M. B. Cosgrave E. M. Killakey E.. (2008). Validation of “prodromal” criteria to detect individuals at ultra high risk of psychosis: 2 year follow-up. Schizophr. Res. 105, 10–17. 10.1016/j.schres.2008.07.012 18765167

[B93] Yung A. R. Phillips L. J. Yuen H. P. McGorry P. D. (2004). Risk factors for psychosis in an ultra high-risk group: psychopathology and clinical features. Schizophr. Res. 67, 131–142. 10.1016/S0920-9964(03)00192-0 14984872

[B94] Yung A. R. Yuen H. P. McGorry P. D. Phillips L. J. Kelly D. Dell'Olio M.. (2005). Mapping the onset of psychosis: the comprehensive assessment of at-risk mental states. Aust. N. Z. J. Psychiatry. 39, 964–971. 10.1080/j.1440-1614.2005.01714.x 16343296

[B95] Zheng L. Wang J. Zhang T. Li H. Li C. Jiang K. (2012). Reliability and validity of the Chinese version of scale of psychosis-risk symptoms. Chin. Mental Health J. 26, 571–576. 10.1111/j.1600-0447.1991.tb03161.x 1746289

